# Fast sequences MR imaging at the investigation of painful skeletal sites in patients with hip osteonecrosis

**DOI:** 10.1186/2193-1801-4-3

**Published:** 2015-01-06

**Authors:** Aristidis H Zibis, Sokratis E Varitimidis, Zoe H Dailiana, Apostolos H Karantanas, Dimitrios L Arvanitis, Konstantinos N Malizos

**Affiliations:** 6 Department of Anatomy Facutly of Medicine, School of Health Sciences, University of Thessaly Panepistimiou 3 (Biopolis), Larissa, 41500 Greece; 7 Department of Orthopaedic Surgery Facutly of Medicine, School of Health Sciences, University of Thessaly Panepistimiou 3 (Biopolis), Larissa, 41500 Greece; 8 Department of Radiology, University Hospital of Heraklion, Heraklion, Crete, 71110 Greece

**Keywords:** Multifocal osteonecrosis, Hip osteonecrosis, Fast MRI sequences, Osteonecrosis

## Abstract

**Background:**

Multiple osteonecrotic foci can be clinically silent when located in metaphyses and becomes painful when it affects juxta-articular areas. The purpose of this study was to assess the value of fast MR imaging to depict the underlying pathology in cases with skeletal pain other than the already diagnosed hip osteonecrosis.

**Methods/design:**

Between 2008 and 2013, 49 patients with already diagnosed hip osteonecrosis reported symptoms of deep skeletal pain in an anatomical site different from the affected hip joint. All patients after thorough history & clinical examination underwent evaluation with x-rays and a single fat suppressed sequence with MR Imaging applying either T2-w TSE or STIR-TSE at the painful site. False positive and false negative findings were recorded for the conventional x-rays and compared to MRI.

**Discussion:**

Forty four (89.8%) patients were positive for osteonecrotic lesions in this study and 76 symptomatic osteonecrosis lesions were revealed at 14 distinct anatomic sites. The agreement between the x-ray findings and the MR imaging regarding osteonecrosis was 46.9%. Plain x-rays showed 43.4% sensitivity, 100% specificity, 100% positive predictive value and 10.4% negative predictive value.

Fast MR imaging with fat suppressed sequences is necessary and adequate as a single method for the investigation of painful skeletal sites in patients with already diagnosed hip osteonecrosis. It allows early diagnosis of the potentially debilitating multiple juxta-articular lesions and consequently their prompt management.

## Introduction

Osteonecrosis (ON) is defined as the in situ death of all the organic components of a bone segment (Lee et al. 2010). Femoral head is the most commonly affected site of the skeleton with approximately 20,000 new cases reported each year in the United States and 11.500 cases annually in Japan (Aaron 1998; Babis et al. 2011; Mont & Hungerford 1995). ON is a clinical entity affecting young individuals in their 30s and early 40s (Plakseychuk et al. 2001). The natural history of juxta-articular lesions depends on the location, size and extension of the lesion and follows a number of distinct stages, ultimately resulting in destruction of the articular surface of the affected joint thus debilitating the patient (Mont & Hungerford 1995; Collaborative Osteonecrosis Group 1999; Ficat & Arlet 1977; Hungerford 2002; Sugano et al. 1994).

Non-traumatic ON may also affect multiple skeletal sites (Collaborative Osteonecrosis Group 1999; Rostom et al. 2008). There are two major types of ON: medullary bone infarction, involving the trabecular architecture and marrow cavities in metaphyseal sites which is in most cases clinically silent; and the juxta-articular infarction, which are located at the subchondral bone at major joints. The disease in the majority of the patients is affecting the hip joint followed by the shoulder, the knee, the ankle and less commonly the elbow and wrist (Lee et al. 2010; Collaborative Osteonecrosis Group 1999). As most of the symptomatic lesions are sub-articular or para-articular, the weight bearing in daily activities may subsequently lead to fatigue failure of the infarct and articular surface collapse. An early diagnosis and immediate intervention is therefore very important and might preserve the joint from collapse with a variety of joint salvage procedures. Since most of the patients are young, a diagnostic test skipping radiation burden would be clinically useful.

Any attempt to depict an osteonecrotic lesion should use MRImages with adequate fat suppression, since the contrast of the lesion against the low signal intensity of the bone marrow is high. Applying a larger field of view and lower acquisition matrix values, the examination time might be significantly reduced. Therefore, at the presentation of a patient with a painful hip and musculoskeletal pain other than that at the buttock, hip and thigh area, 2–3 additional anatomical sites can be screened at the same time as the one subjected to a detailed MRI scan.

The purpose of this study was to assess the value of a fast MR imaging protocol for the multi-site investigation of patients with femoral head ON, who present with skeletal pain in sites other than the known osteonecrosis at the hip joint.

## Materials and methods

Between 2008 and 2013, 164 patients with non-traumatic femoral head ON were included to the present prospective case series. The study population included referral cases of patients presented after the hip ON had been already diagnosed. All these patients were underwent to a regular follow up every six months. The diagnosis of hip osteonecrosis was performed with combination of conventional x-rays and MRI. When the patient had a operatively treatment at his hip the diagnosis was confirmed with Histology examination. However the histology examination was not one of the diagnostic criteria in this study.

At the initial consultation the patients were informed about the possibility of having more sites of the skeleton affected other than the hip joints and the importance to have be appropriately investigated with an MRI as the most sensitive diagnostic tool for ON. After a detailed description of the evaluation protocol all 49 patients who presented with skeletal pain in locations other than the hip joint were included to the study and consented to undergo investigation with plain x-rays (anteroposterior and lateral views) and fast sequence MR imaging of the clinically relevant sites. The MR imaging examinations were performed with a 1.0-T MR scanner (Intera NT; Philips Medical Systems, Best, The Netherlands) by applying a fat suppressed turbo spin-echo (TSE) T2-W (TR/TE: 3000/90 ms, matrix: 256X256, ETL: 7, slice thickness: 5 mm, six signals acquired) with a duration <5 min or a Turbo-STIR sequence (TR/TE: 3325/90 ms, matrix 320×512, slice thickness: 5 mm, FOV: 320, TSE:12, NSA: 4) with a duration of 5 min. The turbo-STIR sequence is not sensitive to magnetic susceptibility artefacts and therefore it was particularly useful in cases of previous operation in the area of interest, where metallic implants might downgrade the quality of the image.

Our study has been approved by the ethics committee of our university hospital and all subjects have signed an informed consent according to the Health Insurance Portability and Accountability Act (HIPAA).

The evaluation of the imaging data was performed in a consensus basis by a radiologist, with 27 years of experience on musculoskeletal radiology and an orthopaedic surgeon, experienced in the diagnosis and treatment of osteonecrosis. The plain radiographs were evaluated in the beginning in a blind mode followed by a MR imaging one week later. MR imaging was considered as the gold standard for the evaluation of ON lesions. The “band-like” pattern or the “serpentine-like” geographic border, were considered as pathognomonic of subarticular ON and intramedullary infarcts respectively, regardless of the applied sequence (Karantanas 2007; Malizos et al. 2007).

In all patients, every single site with skeletal pain was recorded and examined both with plain radiographs and MR imaging. The sensitivity, specificity, positive and negative predictive values were estimated for the plain radiographs. Sensitivity and specificity were calculated according to regular forms after obtaining the true and false positive and negative results. No specific statistical tests were used as the figures are just descriptive (i.e. percentages, number of lesions etc.) MR imaging was considered the standard of reference.

## Result

After clinical evaluation, 49 of the 164 patients (29.8%) underwent the investigation process with the described imaging protocol. Eighty one symptomatic sites were examined with a fast MRI sequence and 76 (94%) of the sites revealed an ON lesion with 67.1% of them being sub-articular or para-articular. Five painful sites (6%) did not show any ON lesions or any other pathology with fast MRI. These five cases during the semester follow up were evaluated and did not complain again for the pain in these specific sites. They never diagnosed with osteonecrosis in these specific sites. In forty four of the 49 symptomatic patients (89.8%) osteonecrotic lesions were depicted with a fast sequence MR imaging in locations other than the femoral heads. There were 32 males (72.7%) and 12 females (27.3%) with a mean age at presentation of 36.4 years (range, 21–65 years). Among these patients 32 (72.7%) presented with bilateral femoral head ON and 12 (27.3%) with unilateral (76 hips affected). In 78.5% of the patients, ON was secondary and in 21.5% of the patients was idiopathic.

All of the positive MRI exams revealed lesions corresponding to the painful sites indicated by the patients. In total, an additional 76 symptomatic ON lesions (excluding the contralateral hip lesions), were recorded in 44 patients (1.7 lesions per patient) at 14 distinct anatomic sites (Table 1). The sites most commonly indicated as painful by the patients were the humeral head (21 of 81) and the acetabulum (17 of 81) (Table 1). All painful acetabular sites and the majority of humeral head sites had positive MR imaging findings whereas less than half of the lesions were depicted on the x-rays. The agreement between the x-rays and the MR imaging was 46.9% (Table 1). The plain x-rays showed 43.4% sensitivity, 100% specificity, 100% positive predictive value and 10.4% negative predictive value 10.4%.

**Table 1:** **True positive and false negative results for the evaluation of the osteonecrotic lesion with X-rays**

	Location	Complains of symptomatic sites	ON lesions MRI	X-rays	
				True positive	False negative
1	Humeral Head	21	18	14	4
2	Humeral Condyles	2	2	1	1
3	Acetabulum	17	17	1	16
4	Sacrum	1	1	0	1
5	Pubic Bone	1	1	0	1
6	Ilium	4	4	0	4
7	Ischial Tuberocity	4	4	0	4
8	Femur	9	9	7	2
9	Femur Condyles	7	7	5	2
10	Tibia Plateau	2	2	0	2
11	Patella	1	1	1	0
12	Tibia	8	6	3	3
13	Talus	3	3	1	2
14	Navicular	1	1	0	1
	Total	81	76	33	43

Thirty one of the 76 newly detected osteonecrotic lesions (40.8%) were subsequently operated and the rest 45 (59.2%) were followed with observation with a conservative management.

## Discussion

Osteonecrosis is a clinical entity most commonly affecting the femoral head, but it may also affect other sites of the skeleton further disabling the patients (Collaborative Osteonecrosis Group 1999). There are two major types of bone infracts in the skeleton. The intramedullary infarctions which are often silent, and those where both the cancellous bone and the subchondral plate are involved in sub-articular or para-articular sites (Lee et al. 2010; Collaborative Osteonecrosis Group 1999; Rajpura et al. 2011). The latter, under the load bearing of the articular surface in daily living, undergo weakening of the affected bone by the osteoclasts and the lack of repair, gradually collapse and become painful finally leading to joint destruction. For this reason the early investigation and treatment of skeletal pain in patients with known femoral head ON is very important. The fast MR imaging is an accurate and reliable diagnostic tool for following up patients with osteonecrosis.

In accordance with Jones et al. MR imaging has a high sensitivity and specificity in the diagnosis of ON and should be the imaging modality of choice when suspicion arises after the clinical evaluation (Jones 1994). MR imaging is the most sensitive imaging modality in the detection of ON and has relatively specific findings (Jones 1994). Evaluation with an efficient MR imaging sequence can be very fast at a low cost. Because of the inherent presence of fat in the bone marrow, such a sequence should be able to adequately suppress the signal from fat, such as the T2-w TSE with spectral pre-saturation or the STIR. Another significant advantage for the fast MR imaging evaluation is that the patient is not exposed to irradiation. As most of the patients in the study suffered from secondary ON they have already had undergone x-rays, bone scintigraphy and occasionally computed tomography in the course of the underline disease, diagnosis and management with considerable irradiation exposure. Whole-body MR imaging examination with a moving table is now available in modern scanners and it offers a more comprehensive modality in the diagnostic evaluation of ON patients (Sakai et al. 2002).

In the present study the imaging modalities applied for evaluation of the skeletal pain in patients with diagnosed ON of the hip included x-rays in two planes and fast sequence MR imaging. Plain radiographs show a poor diagnostic accuracy in medullary disorders, including osteonecrosis (Sakai et al. 2001). However, to the best of our knowledge, their accuracy has never been reported with regard to multifocal osteonecrosis. In addition, a basic evaluation with plain radiographs is important in most patients with skeletal pain, to rule out other disorders than the expected one secondary to the underlying pathology.

It is well documented that bone scan scintigraphy with Technetium 99 has several advantages including the ability to image the entire skeleton at one time, and thus to identify multiple skeletal lesions. It can also be used in patients with cardiac pacemakers, intracranial clips, and claustrophobia, which cannot undergo MR imaging examination (Sakai et al. 2002). On the other hand bone scintigraphy is a method with significantly lower sensitivity, specificity and accuracy compared with the MR imaging, irradiating the patient at the same time which is a matter of concern when evaluating young patients. For the ON of femoral head, the Tc99m bone scan presents 77.5% sensitivity, 75% specificity and 76% accuracy as opposed to 88.8%, 100% and 94% for the MR imaging respectively (Karantanas & Drakonaki 2011). Sakai et al. used MR imaging as the gold standard and reported sensitivity, specificity and accuracy of bone scan for shoulder ON 65%, 81%, 77% and for the knee ON 63%, 71%, 68% respectively (Sakai et al. 2001; Sakai et al. 2002). Finally, it has been demonstrated that bone scans may miss osteonecrotic lesions in 10% to 20% and may not be the best diagnostic modality in these patients (Mont et al. 1997). Due to the irradiation exposure and the reasons described above, bone scintigraphy was excluded as an evaluation method in the present study.

When osteonecrosis affects three or more anatomic sites is defined as multifocal (Collaborative Osteonecrosis Group 1999). The incidence of multifocal ON in the majority of cases is depended on the etiology. Corticosteroid administration, connective tissue disorders, dysbarism, hemoglobinopathies, arteritis/vasculitis, pancreatitis, Gaucher's disease, pregnancy, antiphospholipid antibody syndrome and alcohol and tobacco overuse demonstrate a strong correlation with multifocal ON (Lee et al. 2010; Tektonidou et al. 2003). Multiple-site lesions have also been reported in patients with HIV infection as well as in patients who have undergone renal, cardiac or bone marrow transplantation and in oncologic patients who have received chemotherapy and/or radiation therapy (Tauchmanovà et al. 2003; Mullan & Ryan 2002; Mascarin 1991; Hedri et al. 2007). However, 85-91% of patients with multifocal ON have a history of high intake of corticosteroids (Collaborative Osteonecrosis Group 1999; Jones 1994; Powell et al. 2010). Multifocal ON has an incidence of 5-15% in patients with already diagnosed femoral head ON, 40% in patients already diagnosed with knee ON, 57% of patients with ankle ON and 60% in patients with shoulder ON (Mont et al. 2008). These findings emphasize the necessity of high index of suspicion for any complaints of skeletal pain in patients with ON of the hip, knee, shoulder, or ankle because such patients have higher possibility of being involved by a multifocal juxta-articular disease.

In the current study, the majority of the patients (78.5%) suffered from secondary ON with 67.1% of the lesions being sub-articular or para-articular. Early diagnosis and treatment is essential for any juxta-articular ON lesion in order to prevent irreversible bone and joint destruction, especially when a major joint like the hip has been already affected and co-exists with underlying disease(s) in the same patients. For the small ON lesions without articular surface collapse or at the beginning of their course for non articular lesions high-energy shock wave, statin administration or enoxaparin has been proposed (Kerachian et al. 2006; Li et al. 2003; Wang et al. 2011; Pritchett 2001). For these reasons the need for a high index of suspicion for every painful area in patients with secondary hip ON, especially when there was a previous treatment with corticosteroids, must be emphasized in order to achieve early diagnosis and treatment. This is very important since the long term survival of joint replacement in young patients with hip and knee osteonecrosis have been less than optimal (Mont & Hungerford 1995; Sakai et al. 2002).

**Figure 1:**
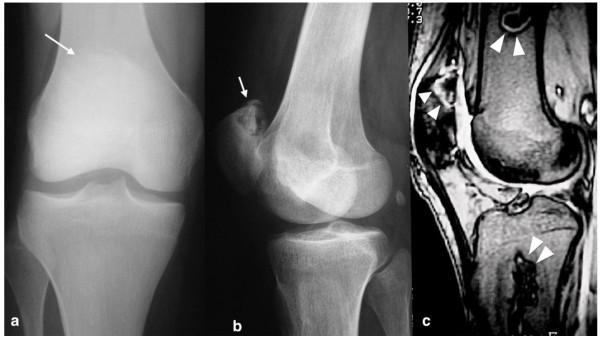
**A 33-year-old patient with history of S.L.E. and hip ON, presented with a painful knee.** The plain x-rays **(a,b)** revealed an ostenecrosis lesion at the superior pole of the patella (arrows). The sagittal fat suppressed T2-w TSE MRI **(c)** showed this ON lesion at the patella and two additional lesions at femur and tibia (arrowheads).

**Figure 2:**
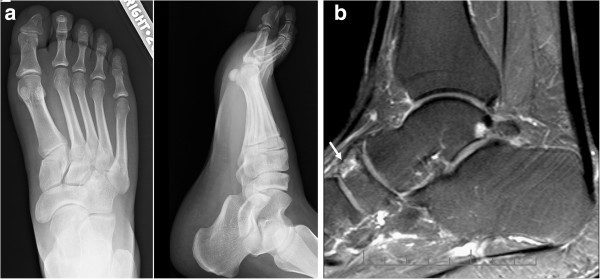
**A 48-year-old patient with a history of asthma and hip osteonecrosis presented with a painful ankle.** The plain x-rays **(a, b)** were negative. The sagittal fat suppressed T2-w TSE MR image **(c)** showed a small ON lesion in the navicular (arrow).

**Figure 3:**
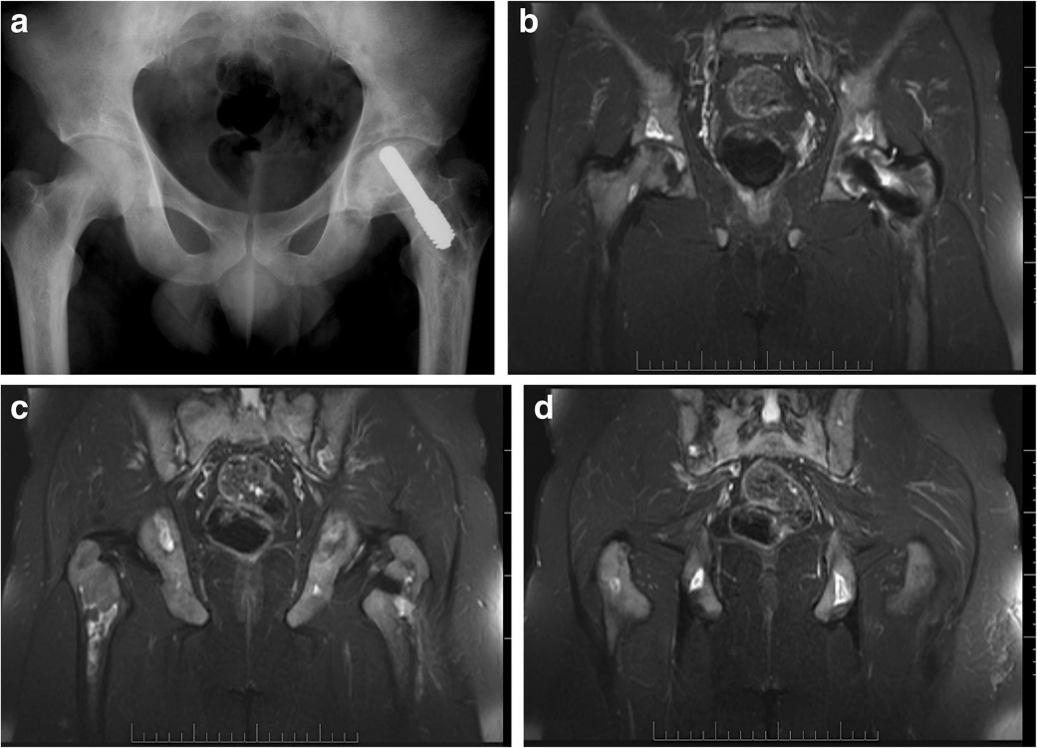
**A 23-year-old patient with a history of hemoglobinopathy (B-thallasaemia) and osteonecrosis of the left hip, which was treated with porous tantalium rod implantation.** The patient presented at the follow up examination with multiple painful sites in the pelvis. Plain AP x-ray of the pelvis was negative **(a)**. The coronal fat suppressed T2-w TSE images **(b-d)** depicted multiple bone infarcts bilaterally at the ischiac bones, acetabuli and proximal femoral diaphyses.

**Figure 4:**
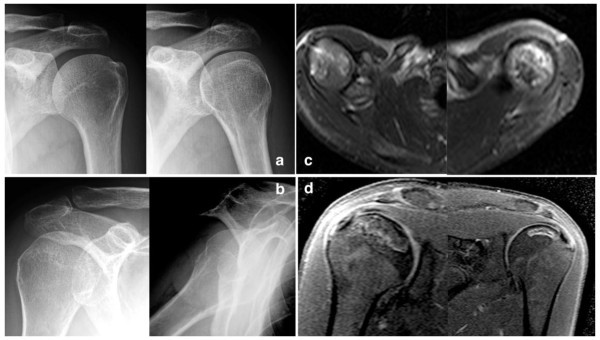
**A 28-year-old patient with a history of acute lymphoblastic leukemia and hip osteonecrosis presented with painful shoulders.** The plain x-rays **(a, b)** were negative. The axial fat suppressed T2-w TSE MR images **(c)** showed a small ON lesion in both humerus head. Because of the severity symptoms it was decided to undergo to the surgical treatment with core decompression. For preoperative planning reasons he underwent to a typical MRI (T1-W STIR) in order to have a more accurate evaluations of the lesions size **(d)**.

Plain x-rays as an investigation exam in the present study, showed 43.4% sensitivity, 100% specificity, 100% positive predictive value and a negative predictive value of 10.4%. Therefore, patients with negative plain radiographs should undergo an MR imaging to confirm the clinical diagnosis (Figures 1, 2, 3, 4). The patients of the present study were young (mean age 36.4 years old) and active and most of them suffered from a secondary ON. Therefore, an early and accurate diagnosis of all affected sites with just one imaging method would be ideal.

A limitation of the present study is that a direct comparison between the two sequences was not performed. This was not possible in many patients as the STIR sequence was selected in patients with metallic implants in whom the T2-w TSE suffers from susceptibility artifacts and cannot be applied.

In conclusion fast MR imaging is an accurate and reliable diagnostic tool for the investigation of skeletal pain in patients with known femoral head ON. Fast MR imaging is particularly helpful for patients with hip ON of secondary etiology where a considerable possibility for multiple ON lesions exists. The fat suppressed T2-w TSE sequence can be applied as a reliable investigation exam and as the single most sensitive and specific diagnostic tool in patients with osteonecrosis.
